# Revisiting dose-finding of monoclonal antibodies in migraine

**DOI:** 10.1186/s10194-023-01602-4

**Published:** 2023-06-09

**Authors:** Linda Al-Hassany, Nazia Karsan, Christian Lampl, Peter J. Goadsby, Antoinette MaassenVanDenBrink

**Affiliations:** 1 Division of Vascular Medicine and Pharmacology, Department of Internal Medicine, Erasmus MC, University Medical Center Rotterdam, PO Box 2040, 3000 CA Rotterdam, The Netherlands; 2 NIHR SLaM Clinical Research Facility, King’s College London, London, UK; 3 Department of Neurology, University of California, Los Angeles, Los Angeles, CA USA; 4 Department of Neurology and Stroke Unit, Koventhospital Barmherzige Brüder Linz, Linz, Austria; 5 Headache Medical Center Linz, Linz, Austria

**Keywords:** Migraine, Monoclonal antibodies, Dose-finding, Dose-ranging, Phase I Clinical Trials, Early phase II clinical trials

## Abstract

Migraine is a debilitating disorder, and while the introduction of monoclonal antibodies (mAbs) has led to efficacious and tolerable responses, a substantial number of patients are so-called “non-responders”. We introduce reasons for this insufficient response, including insufficient blockade of Calcitonin Gene-Related Peptide (CGRP) or its receptor. We present a clinical case, i.e. a female migraine patient who mistakenly administered supratherapeutic (three-fold higher) doses of erenumab leading to more efficacious clinical responses without any side-effects. This example illustrates that the initial dosages might have been too low, resulting in a remaining undesired increased effect of CGRP. While a capsaicin forearm model has repeatedly been used to evaluate the pharmacokinetic-pharmacodynamic relationship of mAbs, we provide directions to revisit or reconsider dose-finding and dose-ranging of these drugs. These directions include (i) refinement and application of a capsaicin forehead model (instead of a forearm model) to study trigeminovascular activity and improve dosing, and (ii) reconsideration of trial populations. Indeed, the dose-finding studies were mainly performed in relatively young and normal-weight males, while most phase III/IV trials are marked by a high female-to-male ratio, mainly consisting of overweight to obese females. Considering these aspects in future trials could optimize healthcare for a larger proportion of migraine patients.

Migraine is a neurovascular disorder characterized by incapacitating headache attacks accompanied by central symptoms, including nausea, vomiting, photo- and phonophobia. Migraine is a major contributor to disability globally and the first cause of disability in women under the age of fifty, in whom the prevalence is the highest. Although the exact pathophysiology of migraine remains elusive, the neuropeptide Calcitonin Gene-Related Peptide (CGRP) has consistently been confirmed to play a pivotal role in migraine [1]. The development of monoclonal antibodies (mAbs) targeting the peptide CGRP (eptinezumab, fremanezumab, and galcanezumab) or its receptor (erenumab) heralds a revolutionary era in the preventive management of migraine. While these mAbs have proven to be efficacious, tolerable, and safe for the treatment of episodic and chronic migraine [1], a substantial proportion (15–25%) of migraine patients treated with these antibodies terminate their therapy due to the lack of efficacy and are so-called “non-responders” [2]. Further, even in chronic migraine patients, who, according to standardized outcome measures of efficacy (such as monthly migraine days, MMD), show a relatively good response to mAbs compared to baseline, the absolute residual migraine burden remains high [3]. It is self-evident that this has far-reaching repercussions on the quality of life and burden in migraine patients, often accompanied by overuse of acutely acting antimigraine drugs, as well as increased costs related to healthcare resource utilization [4].

We propose three hypotheses contributing to inadequate or insufficient response to antibodies in this non-negligible subgroup of migraine patients, namely: (i) blockade of CGRP or its receptor is insufficient; (ii) blockade of CGRP or its receptor is sufficient, but other peptides (e.g. adrenomedullin) can bind to the CGRP receptor, or CGRP can exert its effect through binding to other receptors (e.g. amylin receptors); (iii) blockade of CGRP or its receptor is sufficient, but migraine is induced via alternative non-CGRP-mediated pathways (e.g. through the pituitary adenylate cyclase-activating polypeptide (PACAP) pathway) [1]. Based on current knowledge and treatment options, testing the second and third hypotheses is impractical, although interestingly switching from erenumab to a CGRP-targeted antibody has recently been proven to be a pragmatic approach in those failing treatment with erenumab [2]. An overlooked aspect related to the first hypothesis concerns the clinically approved dosages of mAbs. With the introduction of a clinical case and a discussion about different human experimental models, we aim to provide some early directions on why and how we should revisit or reconsider dose-finding and dose-ranging in current phase I and early phase II clinical trials on mAbs in migraine.

A 54-year-old woman with a 20-year history of chronic migraine and medication overuse was referred to the tertiary Headache Clinic at King’s College Hospital, London, for headache worsening associated with the perimenopause. Her BMI was 22. She complained of daily continuous headache, with eight migraine days a month. She used a combination analgesic containing paracetamol and codeine daily and rizatriptan 2 days a month. A small dose of amitriptyline 10 mg was mildly useful; she failed to tolerate higher doses due to cognitive side effects. Propranolol and Botox therapy had been tried before without effect. Erenumab treatment was commenced in January 2019, initially at 70 mg per month, and at 140 mg (administered as 2 × 70 mg doses) after 6 months, supplied via the Novartis free-of-charge scheme. This scheme provided erenumab to some responders following the clinical trials pending UK licensing. There was a reduction in headache severity following the first 70 mg dose (50%), which was sustained but more dramatic on the 140 mg dose (80%), with an associated reduction in migraine-associated nausea and vomiting, but no change in headache or migraine frequency. There was a 50% reduction in analgesic use. The MIDAS (Migraine Disability Assessment Test) score reduced from 121 to 8 following a year of treatment compared to baseline. There were no adverse effects.

In October and November 2021, she was sent deliveries of 3-month batches of the 140 mg pre-filled injection pens each month, following NHS approval for the 140 mg single dose only. She mistakenly administered all three 140 mg injections received (total dose of 420 mg/month) for these two consecutive months. There were no complications or side effects. Following medical advice from our team, she had a treatment break for 3 months and resumed 140 mg monthly in February 2022. By the end of November 2021, she had stopped taking paracetamol and codeine entirely, with a sustained benefit on review in March 2022, with twenty headache days a month (33% reduction) and eight migraine days, eight triptan days and no paracetamol or opioid days (73% reduction in medication days). According to the UK NICE guidelines, this is an adequate response to continue treatment. Blood pressure and routine blood tests including full blood count, renal and liver profiles were normal following this event, other than a lymphopenia of 0.6, which was also present on historical blood tests and was therefore an incidental finding.

Based on this clinical case, it can be concluded that the initial dosage prescribed to this migraine patient might have been too low, resulting in insufficient blockade of the CGRP receptor and a remaining undesired increased effect of CGRP. Following in vitro binding studies, approved dosages of mAbs in migraine, including erenumab, are mostly based on a non-invasive and safe human pharmacodynamic model**,** where capsaicin is applied on the volar forearm [5]. This causes local neurogenic inflammation and vasodilation, primarily mediated by CGRP through the activation of transient receptor potential vanilloid type 1 (TRPV1) receptors. The increase in dermal blood flow (DBF) is measured by laser Doppler flowmetry and allows an assessment of responses to CGRP(-receptor) blockade by serving as a target engagement biomarker. The capsaicin forearm model has been repeatedly used to evaluate the safety, tolerability and pharmacokinetic-pharmacodynamic relationship of erenumab [6, 7] and galcanezumab [8, 9] – serving as a guide for dose selection in clinical trials [10].

Despite the reproducible results of early-phase clinical trials using the capsaicin forearm model, we warrant caution in translating these findings on dosages of mAbs to desired clinical responses of migraine patients in phase III and IV (real-world) studies. Our greatest concern is that changes in the forearm microvascular circulation are not necessarily representative of changes in the trigeminovascular system. This system is specifically studied in our capsaicin forehead model, which has been validated [11] and can detect variations in trigeminovascular reactivity that depend on changes in the menstrual cycle [12]. More recently, it was shown that migraine patients with a good response to erenumab (≥ 50% reduction in MMDs) differ from patients with a poorer response (< 50% reduction in MMDs), based on a lower initial trigeminovascular activity [13]. In this model, 70 mg erenumab produced an inhibition of approximately 50% of the capsaicin-induced response [13], while in the forearm model, using equivalent doses of capsaicin, 70 mg erenumab led to a maximum DBF inhibition of approximately 90% [6]. Moreover, higher doses of 140 mg and 210 mg did not lead to further inhibition [6], which seems to be discordant with the greater clinical treatment effects for 140 mg versus 70 mg erenumab, at least in (difficult-to-treat) chronic migraine patients in whom prior preventive treatment had failed [14]. Thus, the capsaicin model might indicate different doses of CGRP(-receptor) blockers as optimal, depending on the location (trigeminovascular or peripheral) of the measurements (Fig. 1). It remains to be demonstrated whether this could be alternatively explained by a different response of cutaneous tissue overlying the soft tissue of the forearm *versus* the skin overlying the bone in the forehead – either due to actions of capsaicin or to CGRP. In addition, while both models measure capsaicin-induced DBF, they do not provide information on different non-vascular effects of CGRP in migraine, e.g. its actions on macrophages [15]. Therefore, further studies are warranted to understand these alternative effects of CGRP as well as its interactions with other systems (e.g. hormones and other neuropeptides), and their implications for drug testing models.

**Fig. 1:**
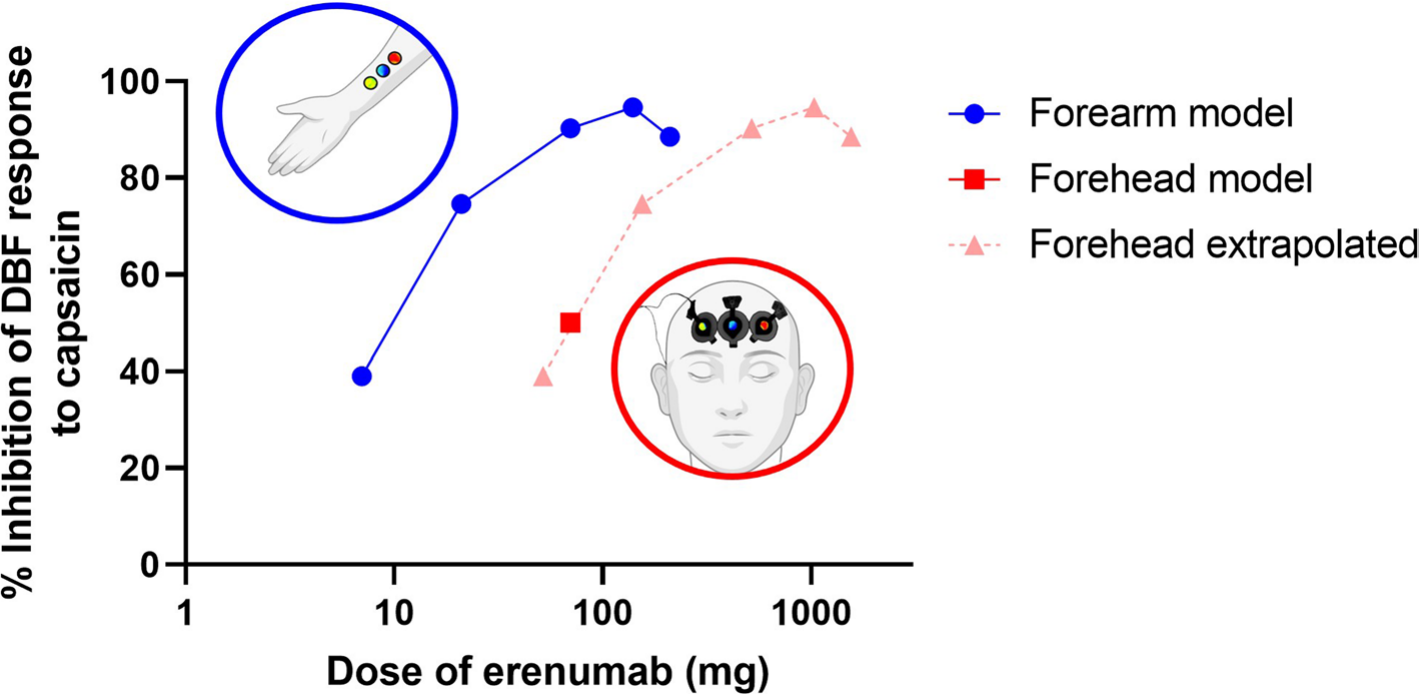
Representation of the percentage inhibition of DBF in response to capsaicin application on the forearm model with increasing doses of erenumab (blue graph, [6]). Based on a study in the forehead model using 70 mg of erenumab (red square, [13]), we extrapolated the forehead DBF responses to a wider range of dosages (pink graph)

An additional concern relates to the study populations, as the capsaicin forearm model was studied mainly in (relatively young) males [6–9]. Importantly, hormonal influences were observed in healthy females, especially during menstruation, and have been demonstrated to increase DBF responses to capsaicin [10, 16]. An explanation might be the increased release of CGRP in women [17]. Therefore, one could hypothesize that the dosages used in the male populations of the capsaicin forearm model studies are insufficient to block the higher CGRP levels in female migraine patients. This is of importance given the female predominance of migraine and the high female-to-male ratio in phase III/IV trials. Further, as described in the validation study, a proportion of participants was excluded as they did not respond to capsaicin [5]. This might have led to unjustified selection bias in early trials, as we have previously shown that applying iontophoresis of capsaicin in these “non-responders” in the forearm model leads to “responders” in the forehead model (unpublished results, [11]). Lastly, while the BMI of (healthy) male study populations included in phase I trials lie in the normal range, the BMI of older females included in phase III/IV studies, primarily performed in the United States of America, might be higher [18]. Body weight might influence the pharmacokinetics of mAbs, especially galcanezumab [18, 19], and probably also their efficacy in certain obese patients based on our clinical experience. Although an inverse relationship between body weight and efficacy cannot be proven yet, we have several morbidly obese patients, who are unresponsive to any of the available antibodies. This is of clinical relevance especially given the often-reported bidirectional relationship between migraine and obesity, of which prevalence is rising globally. The association between both conditions is probably related to the increased activity and mutual role of CGRP [20]. Therefore, DBF responses after blocking CGRP activity in healthy males might not be translatable to responses in migraine patients with a higher BMI in real-world settings.

While the abovementioned clinical features might have subtle individual effects, we highlight their synergistic effects when present in combination [18], leading to suboptimal or absent responses to mAbs when used in a substantially other (target) population than research populations on which their dosages were based upon.

In conclusion, we encourage further research and the use of the forehead capsaicin model as an appropriate model to study trigeminovascular activity, and further refinement of the model for its application in dose-finding trials. Further, we advocate for choosing a representative study population in dose-finding trials to avoid a suboptimal choice for a clinical dose in migraine patients. Indeed, subtherapeutic doses might be used in clinical practice in some patients, resulting in insufficient blockade of CGRP activity.

Obviously, with the introduction of a single clinical case, no implications can be drawn yet on mAb underdosing in all migraine patients. Double-blind studies are warranted to demonstrate whether e.g. doubling of a therapeutic dose leads to better efficacy in specific patients, and basic animal research should provide insight into the underlying mechanisms. Here, we primarily provide a critical reappraisal to review the current conduction of dose-finding clinical trials on mAbs in migraine in which drug companies use the capsaicin forearm model as one-step of the developmental program. Admittedly, current clinical trial data on galcanezumab [21] and fremanezumab [22] do not directly imply that higher doses directly yield better endpoints (i.e. reduction in MMDs), but the proportion of non-responders is substantial. Further, translating drug responses from clinical trial populations to population-based settings is inevitably accompanied by limitations in the generalizability of trial data (e.g. due to a different pathophysiological mechanisms and patient characteristics). In this context, it is noteworthy that increasing dosages of erenumab have been shown to be beneficial for a substantial proportion of individuals in the real-world setting with almost half of the patients reporting that the dose increase was helpful [23]. Yet, vigilance is still advised, considering the *potential* cardiovascular risk that could accompany long-term blockade of CGRP activity, acting as a potent vasodilator under ischemic conditions, in migraine patients [24], as well as other potential non-cardiovascular side effects [25]. Considering and implementing these aspects in future dose-finding and dose-ranging studies in antimigraine drug trials could optimize healthcare in a large proportion of migraine patients and further decrease the enormous burden of migraine globally.

## Data Availability

Not applicable.

## Abbreviations

BMI: Body Mass Index
CGRP: Calcitonin Gene-Related Peptide
DBF: Dermal Blood Flow
mAb: Monoclonal Antibody
MIDAS: Migraine Disability Assessment Test
MMD: Monthly Migraine Day
PACAP: Pituitary Adenylate Cyclase-Activating Polypeptide
TRPV1: Transient Receptor Potential Vanilloid type 1
